# Supplementary material for: Mechanistic Study of Novel Dipeptidyl Peptidase IV Inhibitory Peptides from Goat’s Milk Based on Peptidomics and In Silico Analysis
Source: Foods. 2024 Apr 14;13(8):1194. doi: 10.3390/foods13081194 (PMC11049645; doi:10.3390/foods13081194)
Supplement: Supplementary file 1 [file foods-13-01194-s001.zip › foods-2920443-supplementary.pdf]

Supplementary Data

A

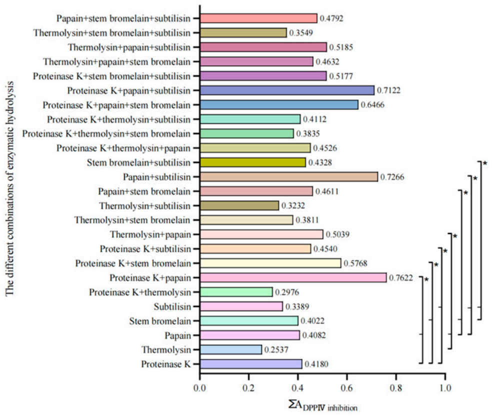

Figure S1. The total frequency of DPP-IV inhibitory peptides (∑A_{DPP-IV} inhibition value) in goat casein and whey protein by *in silico* proteolysis using single enzymes or enzyme combinations. The * represents significant differences among different enzyme combinations ($p < 0.05$).

A B

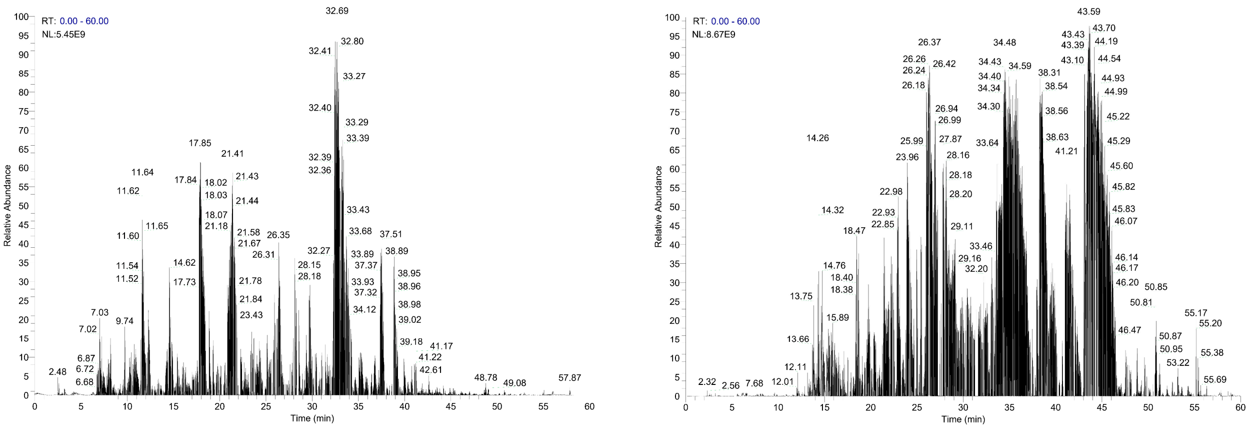

Figure S2. Basepeak plot obtained by LC-MS/MS for casein and whey protein in goat milk. A, basepeak plot of whey protein. B, basepeak plot of casein.

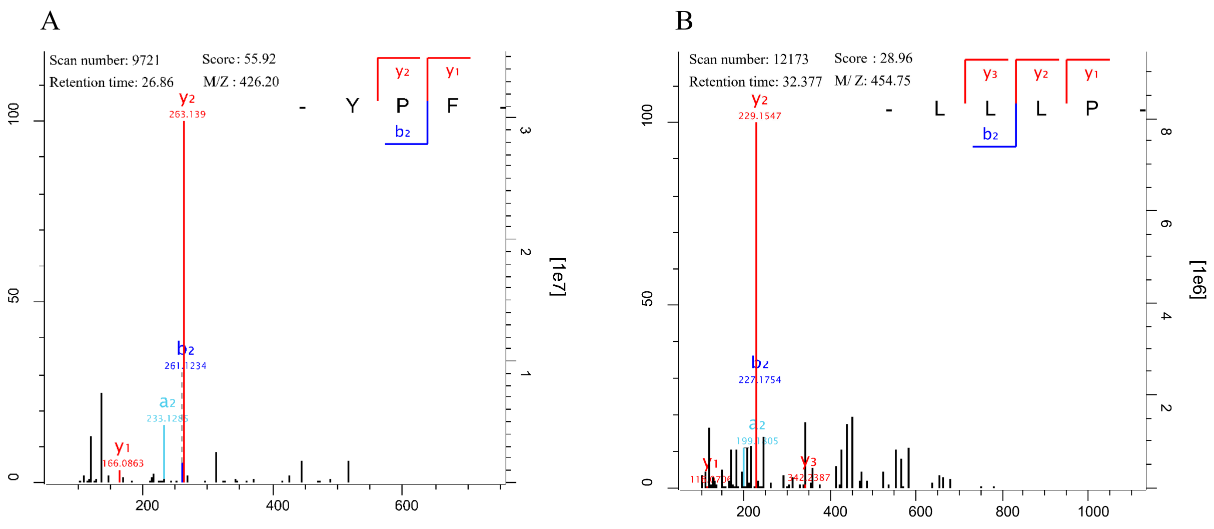

Figure S3. Mass spectrogram obtained by LC-MS/MS for YPF and LLLP in goat milk. A, mass spectrogram of YPF. B, mass spectrogram of LLLP.

Table S1 Source of identified peptides

Number	Whey protein	Casein protein	Whey protein and casein protein
1	RVY	YPF	YPF
2	KYL	WKK	WKK
3	FLF	YLY	YLY
4	WLQ	YLL	YLL
5	VLW	YLM	YLM
6	VVW	YVM	YVM
7	NEW	YVF	YVF
8	LLW	VLW	VLW
9	YLF	WYL	WYL
10	LKF	LFF	LFF
11	LYL	MLY	MLY
12	YVF	LSW	LSW
13	VWF	LEY	LEY
14	WTL	LLW	LLW
15	KLF	HPF	HPF
16	LRL	WSF	WSF
17	WLE	SWL	SWL
18	YLL	FFK	FFK
19	WSL	YAY	YAY
20	WVY	LQW	LQW
21	LEY	YQL	EFR
22	LFW	EFR	LWV
23	WYL	LWV	TLW
24	LFF	TLW	FEL
25	FEL	FEL	VVW
26	FSW	VVW	EYL
27	FLQ	EYL	VFY
28	TLW	VFY	FLF
29	LWF	FLF	LWF
30	VWE	WFK	FLQ
31	YVY	RLF	LYQ
32	RWL	LWF	WTL
33	YVE	FLQ	KDF
34	LVW	LYQ	RFL
35	DYL	WTL	WLQ
36	EYL	SWE	HFL
37	YLY	KDF	VTW
38	KLY	RFL	LWK
39	VYW	WLQ	MFF
40	FVY	HFL	AFY
41	FLK	YYV	DYL
42	WKK	VTW	NLY

Number	Whey protein	Casein protein	Whey protein and casein protein
43	VEW	LWK	YTF
44	YRL	WEV	LLR
45	VFY	MFF	WLM
46	LFE	AFY	YYF
47	HFL	DYL	FEK
48	FFK	YPY	FVY
49	LSW	NLY	TWY
50	LLR	NYY	LEF
51	WDL	YTF	LFW
52	TWY	LLR	KLY
53	HPF	WLM	VEW
54	WLM	TYF	YSF
55	LTW	YYF	TFF
56	LEF	WKP	WAE
57	TFF	FRY	YLR
58	YLE	FEK	FLK
59	VTW	FVY	VWF
60	SWL	TWY	WVL
61	YPF	LEF	YVR
62	WRQ	TWL	YWL
63	YSF	LFW	WVY
64	YVQ	WAY	RWL
65	WVL	FLE	VWY
66	AWY	KLY	QVY
67	PFR	VEW	LRL
68	WQL	YSF	FKF
69	FDF	TFF	LHY
70	YHR	WAE	YFS
71	QDF	YLR	FTR
72	LYQ	WYA	HYL
73	YAY	FLK	YLK
74	YLK	VWF	MEY
75	LHY	SYF	YLE
76	RLQ	WVL	SKW
77	KDF	YVR	YVE
78	RFL	YWL	YRL
79	LHW	WVY	RSF
80	MFF	RWL	NEW
81	NFF	KEY	FNQ
82	TFW	VWY	YYK
83	LQW	QVY	QFE
84	YTF	FEY	WAH
85	HYL	RDW	REL

Number	Whey protein	Casein protein	Whey protein and casein protein
86	TQW	LNY	YVH
87	SKW	LRL	FDQ
88	WRK	FKF	WLH
89	FEK	LHY	RVY
90	SEW	YRY	YHT
91	VMY	WSR	FYK
92	ELF	NWT	HER
93	YHL	YFS	VYPF
94	RFQ	LYR	VPPF
95	WLD	FYA	YLPL
96	YYF	FTR	VTVL
97	WFT	HYL	TLVF
98	LWV	YLK	TLLA
99	VWY	MEY	PEVL
100	LWK	YLE	TSVL
101	WTQ	SKW	LLPL
102	MWL	YVE	PAAF
103	EFR	YRL	TLTL
104	MLY	YYH	VTTL
105	MSW	RSF	YVLA
106	RAW	NEW	VTPQ
107	YYK	FNQ	YVYP
108	RSF	YHV	QLPE
109	WLH	YYK	VTEL
110	QVY	QFE	TLAL
111	KMF	WAH	LLLL
112	LYK	REL	TDVL
113	VWK	YVH	LFSL
114	EFM	FDQ	TPVV
115	TTW	WLH	VTLY
116	FWV	RVY	LLLP
117	YWL	YHT	VSVL
118	YHF	FYK	VKPE
119	FNQ	HER	VTLV
120	PWL	VYPF	YVVT
121	LRQ	VPPF	VLLK
122	YLD	FTVP	LLTV
123	YFS	YLPL	VLTV
124	WAE	FPPQ	VPVQ
125	FKF	VTVL	TLLL
126	AFY	TLVF	VATL
127	VYR	TLLA	VTLF
128	MHF	PEVL	YLAV

Number	Whey protein	Casein protein	Whey protein and casein protein
129	NLY	TSVL	FEVS
130	EDF	LLPL	LLKL
131	YLM	PAYT	VTLQ
132	QFE	PAAF	VTAF
133	YLR	TLTL	YTTL
134	QFF	VTTL	TLTV
135	WAQ	YVLA	TLLT
136	RMF	VTPQ	SLTF
137	TPW	TDVE	RHVP
138	HER	YVYP	TPTK
139	YWS	QLPE	YVLT
140	FYK	TLDV	GEDF
141	KFH	VTEL	SKLL
142	FQF	TLAL	LEPL
143	RDF	LLLL	FEVQ
144	LEW	TDVL	VTVQ
145	YVH	LFSL	VLLT
146	QPY	TPVV	YESL
147	YTM	VTLY	SFAL
148	YVR	VTAL	TLAV
149	YVM	LEYS	VFTV
150	QQF	LLLP	VLVE
151	EEF	VSVL	LELA
152	TVW	VKPE	VELY
153	YRV	VTLV	YVLL
154	FTR	YVVT	YTLV
155	WKE	TQDT	VTEF
156	RRH	VLLK	VTKE
157	RRQ	LLTV	VTLP
158	RHN	ATSY	NETV
159	FDQ	LLLG	LATF
160	HFT	VLTV	YVVE
161	REL	VPVQ	LLVT
162	TDW	TLLL	LAVQ
163	WTN	VATL	PEVK
164	YHT	VTLF	VTKP
165	MEY	FYPD	VPSK
166	RFK	YLAV	LELF
167	EFH	VFVG	VGAW
168	YLQ	FEVS	GELF
169	THW	LLKL	LEGF
170	QFQ	VTLQ	LEVT
171	RQT	VTAF	LKLE

Number	Whey protein	Casein protein	Whey protein and casein protein
172	WAH	LPAF	VLDL
173	WSF	YTTL	LPPL
174	QFH	TLTV	VSTL
175	VTDL	TLLT	TLSW
176	VYPF	LYVP	VTDL
177	LFSL	HLSW	TAYV
178	VESL	SLTF	TPPL
179	VVVL	RHVP	VKVL
180	VPPF	PDPV	VFLN
181	VTVQ	PLLV	VGQL
182	VTPQ	TPTK	VNVK
183	TLLA	YVLT	YLVA
184	VPVQ	GEDF	FALE
185	LLTV	SKLL	TLQA
186	LHLR	LEPL	TLSL
187	VTTL	FEVQ	VEVF
188	VELL	VTVQ	VELV
189	SPPL	VLLT	TLKL
190	WVEA	YESL	YSLQ
191	LLVQ	SFAL	VLEP
192	VEFA	TLAV	NSLL
193	QLPE	VFTV	SKKA
194	LLLL	VLVE	LEVA
195	TLTL	VHVL	TTAE
196	YLAV	TSLM	LLEA
197	LELY	LELA	VEPF
198	TPPL	VELY	VTLK
199	LLKQ	YVLL	VTSV
200	VQLV	YTLV	LAVE
201	NFPA	VTEF	LEGH
202	VTVL	VSLV	NFPA
203	YLPL	VTKE	LHLR
204	TLVF	VTLP	VALK
205	TPTK	YSVS	VELK
206	PLLL	NETV	FEAL
207	TLTV	YEVL	TPPK
208	WLGL	LATF	LKLS
209	VTSL	YVVE	TLVE
210	VQVA	LLVT	VEFA
211	LELF	LRLF	PPLE
212	VEFL	LAVQ	SSVF
213	VTSV	PEVK	FFTK
214	VAPF	VTKP	EDLQ

Number	Whey protein	Casein protein	Whey protein and casein protein
215	TLLT	MLYQ	VQNL
216	LLAN	VELQ	ELYK
217	LLTL	VPSK	VGLD
218	YVLL	LELF	PGKL
219	LLKL	LDVE	SSKV
220	VLTV	VGAW	VTPE
221	VGQL	PFPT	VVPQ
222	LEVL	GELF	VDVT
223	FELL	LMLQ	FAAE
224	THKV	PFSP	TKEP
225	VPDL	VLPL	VEEE
226	TLNL	LEGF	KLLA
227	FEVS	LEVT	LAVR
228	VETD	LKLE	SNVL
229	TEVE	VLDL	SSTF
230	LELK	LPPL	YTGE
231	TLVE	VSTL	VTTE
232	SDLL	VPVT	TDLA
233	VEVL	TLSW	PGLK
234	VTLV	VTDL	VASE
235	VEVT	GQPK	LLTS
236	MELL	TAYV	MFPPQ
237	YSLL	TPPL	TPVVV
238	YTLL	VKVL	PAALE
239	TLLL	VFLN	KEMPFPK
240	VLVE	VGQL	LPAPIVR
241	LKLS	VNVK	RGPFPILV
242	WLVL	YLVA	TEEEKNRLN
243	VTLQ	PPQP	VEELKPTPEGN
244	LAVE	APAY	GLSPEVPNENLLR
245	LELA	PKPA	ELKPTPEGNLEIL
246	LLEA	FALE	-
247	LLVR	HFLY	-
248	VAKL	TLQA	-
249	TSVL	TLSL	-
250	WLVK	PEPE	-
251	VTLF	PKVL	-
252	VLLT	VEVF	-
253	VTTW	VELV	-
254	LEVT	TLVD	-
255	TKPL	TLKL	-
256	RLLK	YSLQ	-
257	VTEF	VMLY	-

Number	Whey protein	Casein protein	Whey protein and casein protein
258	VTEL	VLEP	-
259	TGLL	SSTK	-
260	VVEQ	VPLL	-
261	VVVK	FHVM	-
262	PEAL	NSLL	-
263	FRPD	SKKA	-
264	LLKV	VFVN	-
265	KCSS	LEVA	-
266	TGLE	TETL	-
267	EALL	PLAD	-
268	TLSL	PLDL	-
269	LFVL	TTAE	-
270	HLPL	LLEA	-
271	VTPE	VEPF	-
272	VLLK	VTLK	-
273	PFVT	VTSV	-
274	VTAE	LAVE	-
275	KLLA	LEGH	-
276	LKLE	TQPL	-
277	PEVL	NFPA	-
278	VTKV	TESF	-
279	LEPL	LHLR	-
280	LPPL	VALK	-
281	ELLK	VELK	-
282	LAVT	FEAL	-
283	NELQ	TPPK	-
284	QPPK	LKLS	-
285	VTDW	SPFQ	-
286	EAVF	TLVE	-
287	LLKD	VEFA	-
288	VEVQ	PPLE	-
289	SLTF	SSVF	-
290	FPEV	FFTK	-
291	KGWS	EDLQ	-
292	YLLK	PKCQ	-
293	LEVA	VQNL	-
294	LLVT	ELYK	-
295	TDLL	VGLD	-
296	QFPF	VPYY	-
297	VSPL	PGKL	-
298	PPGR	VTEA	-
299	VTLP	SSKV	-
300	LLLQ	VTPE	-

Number	Whey protein	Casein protein	Whey protein and casein protein
301	FEFL	VVPQ	-
302	VDPL	VDVT	-
303	VFAE	FAAE	-
304	VLNL	TKEP	-
305	YSLQ	VEEE	-
306	SSLL	KLLA	-
307	VQLS	LAVR	-
308	SFLE	VPSR	-
309	VHKL	SNVL	-
310	YRVY	SSTF	-
311	VPSK	YTGE	-
312	LLAE	VTTE	-
313	VTAF	TDLA	-
314	PLVR	PGLK	-
315	PAAF	VASE	-
316	VEVF	LLTS	-
317	YESL	MFPPQ	-
318	VVLE	MPFPK	-
319	VRLQ	TPVVV	-
320	VLDL	VDVPF	-
321	EDLQ	PAALE	-
322	LKLK	KEMPFPK	-
323	VVTL	LPAPIVR	-
324	VEGL	TPVVVPPF	-
325	YVVA	RGPFPILV	-
326	TLAV	EEEKNRLN	-
327	PLVL	TEDELQDK	-
328	YVYP	TEEEKNRLN	-
329	VVTF	HPHPHLSFM	-
330	VDPP	VASLLGLSL	-
331	VKLD	SPEVPNENLL	-
332	TSQL	EKDERFFDDK	-
333	FDVD	SPEVPNENLLR	-
334	TLDP	MNKLLSPIVPQ	-
335	TKLL	FSDIPNPIGSE	-
336	VNFL	VEELKPTPEGN	-
337	VDVT	KDERFFDDKIA	-
338	EELL	KPTPEGNLEIL	-
339	SKLL	EMPFPKYPVEPF	-
340	YAVA	TPVVVPPFLQPE	-
341	QKLL	TEDELQDKIHPF	-
342	TLAL	LSPEVPNENLLR	-
343	YVLA	PTVVVEDLRLCT	-

Number	Whey protein	Casein protein	Whey protein and casein protein
344	RGQF	SLSQPKVLPVPQ	-
345	LHSL	PILVTPSLDPLQ	-
346	TDVL	GLSPEVPNENLLR	-
347	VGAW	KEMPFPKYPVEPF	-
348	REDF	ELKPTPEGNLEIL	-
349	TLVY	HKEMPFPKYPVEPF	-
350	VAFN	-	-
351	TTSL	-	-
352	VELY	-	-
353	TLKL	-	-
354	VKVL	-	-
355	VALE	-	-
356	GELF	-	-
357	VTLY	-	-
358	VQNL	-	-
359	FAAE	-	-
360	LEVS	-	-
361	VFTV	-	-
362	VTVE	-	-
363	TEVK	-	-
364	WLTL	-	-
365	VATL	-	-
366	KRVL	-	-
367	YPSP	-	-
368	PEVK	-	-
369	LLPL	-	-
370	VMLL	-	-
371	VLAW	-	-
372	GFDL	-	-
373	TSLF	-	-
374	VAVL	-	-
375	FEAL	-	-
376	TDLA	-	-
377	TLSW	-	-
378	YEFK	-	-
379	VALL	-	-
380	YLLE	-	-
381	LFSE	-	-
382	SSML	-	-
383	LSDD	-	-
384	GEDF	-	-
385	VSVL	-	-
386	VELV	-	-

Number	Whey protein	Casein protein	Whey protein and casein protein
387	PFAQ	-	-
388	AYLK	-	-
389	FFTK	-	-
390	MKVL	-	-
391	YVVE	-	-
392	VTKP	-	-
393	VALK	-	-
394	TPVV	-	-
395	PAYV	-	-
396	VKSP	-	-
397	TLLW	-	-
398	RSKC	-	-
399	VTVN	-	-
400	VEPF	-	-
401	VKAE	-	-
402	KAYS	-	-
403	VLSL	-	-
404	SPLL	-	-
405	LETK	-	-
406	PFVL	-	-
407	VVPK	-	-
408	YTLV	-	-
409	YSAE	-	-
410	SFAL	-	-
411	LEKG	-	-
412	LLSA	-	-
413	LLTS	-	-
414	QALL	-	-
415	VTWD	-	-
416	LQQN	-	-
417	YVLT	-	-
418	LAVR	-	-
419	DFGL	-	-
420	SSVF	-	-
421	SPVL	-	-
422	VEPL	-	-
423	SSQL	-	-
424	VFTQ	-	-
425	LEGF	-	-
426	VTLK	-	-
427	FALE	-	-
428	TWAR	-	-
429	TFLE	-	-

Number	Whey protein	Casein protein	Whey protein and casein protein
430	VPKP	-	-
431	VQWL	-	-
432	TLMC	-	-
433	VVPQ	-	-
434	FAVP	-	-
435	TPPK	-	-
436	SNVL	-	-
437	AKLL	-	-
438	LFKV	-	-
439	VTWQ	-	-
440	VELK	-	-
441	FALT	-	-
442	QLKF	-	-
443	EAPF	-	-
444	PALF	-	-
445	YTTL	-	-
446	PEKV	-	-
447	VTTE	-	-
448	ELYK	-	-
449	SDVF	-	-
450	YVVT	-	-
451	VEEE	-	-
452	LRPL	-	-
453	NVTV	-	-
454	SSYT	-	-
455	VLEP	-	-
456	PKPK	-	-
457	VTLH	-	-
458	NSLL	-	-
459	TKEP	-	-
460	LATF	-	-
461	VLVS	-	-
462	VAVR	-	-
463	RHVP	-	-
464	LAVQ	-	-
465	TPEP	-	-
466	VTLT	-	-
467	FDVW	-	-
468	VASE	-	-
469	LLLP	-	-
470	VSTL	-	-
471	LEYQ	-	-
472	QKGW	-	-

Number	Whey protein	Casein protein	Whey protein and casein protein
473	YTGE	-	-
474	YGTD	-	-
475	FEVQ	-	-
476	RFVS	-	-
477	LKEY	-	-
478	VGLD	-	-
479	LAWL	-	-
480	YVHE	-	-
481	SRTT	-	-
482	HKVT	-	-
483	QNLE	-	-
484	VPLK	-	-
485	RPAE	-	-
486	VFLN	-	-
487	TLLK	-	-
488	KAVF	-	-
489	PPLE	-	-
490	VGTY	-	-
491	TLKW	-	-
492	YLVA	-	-
493	VMLE	-	-
494	AAQL	-	-
495	EPYA	-	-
496	YAVK	-	-
497	FDTD	-	-
498	TEPP	-	-
499	TKPE	-	-
500	PNLV	-	-
501	VEDE	-	-
502	PGKL	-	-
503	RLAW	-	-
504	GDTY	-	-
505	EAPT	-	-
506	LKPP	-	-
507	PEFH	-	-
508	LETE	-	-
509	LLRA	-	-
510	NYVV	-	-
511	HEAL	-	-
512	PGWL	-	-
513	VKPE	-	-
514	TTVT	-	-
515	TAYV	-	-

Number	Whey protein	Casein protein	Whey protein and casein protein
516	NETV	-	-
517	LLSK	-	-
518	VERR	-	-
519	EDVS	-	-
520	PGLK	-	-
521	VNVK	-	-
522	VTTK	-	-
523	TTAE	-	-
524	VRKQ	-	-
525	LHTS	-	-
526	SSTF	-	-
527	KGTE	-	-
528	SKKA	-	-
529	PLPP	-	-
530	SSVR	-	-
531	VAKF	-	-
532	LRVK	-	-
533	LDHA	-	-
534	VTSK	-	-
535	YVYK	-	-
536	TLQA	-	-
537	EPVL	-	-
538	VAWF	-	-
539	RQGG	-	-
540	LEGH	-	-
541	LVAK	-	-
542	LVRQ	-	-
543	NVRF	-	-
544	VTKE	-	-
545	VETH	-	-
546	SSKV	-	-
547	VELSN	-	-
548	MFPPQ	-	-
549	TLLTV	-	-
550	VEGLE	-	-
551	TPVVV	-	-
552	THKLE	-	-
553	PAALE	-	-
554	VLPLV	-	-
555	VSVPE	-	-
556	LPAPIVR	-	-
557	KEMPFPK	-	-
558	SPRDVLVR	-	-

Number	Whey protein	Casein protein	Whey protein and casein protein
559	HKEMPFPK	-	-
560	SGDNVSVR	-	-
561	REVELEDQ	-	-
562	RGPFPILV	-	-
563	VSDDYVGR	-	-
564	TSSEIDRF	-	-
565	VRTPEVDKE	-	-
566	WGPLPEPGQ	-	-
567	TEEEKNRLN	-	-
568	GQSGKDPNHF	-	-
569	LTEEEKNRLN	-	-
570	VEELKPTPEGN	-	-
571	SGKDPNHFRPA	-	-
572	GNQELPREKYL	-	-
573	AEDPSQDIVER	-	-
574	LNEPEDETHLE	-	-
575	TYPDSESPLTT	-	-
576	KGGYLPPEEIK	-	-
577	VLAPPQEELSK	-	-
578	EVDDTIQTLQQ	-	-
579	ELTDDKNQRFF	-	-
580	EDPSQDIVERN	-	-
581	SPFPVHPGTPE	-	-
582	RQPQNQNPKLPL	-	-
583	APPQEELSKSTL	-	-
584	GPGAPADPGRPA	-	-
585	AHLHTPMYFFISQ	-	-
586	ELKPTPEGNLEIL	-	-
587	VEELKPTPEGNLE	-	-
588	GLSPEVPNENLLR	-	-
589	QADVIGTETLERL	-	-
590	SPRQPQNQNPKLPL	-	-
591	RSPRQPQNQNPKLPL	-	-
592	ELDPAPNDEVDPGTR	-	-
593	VTYPKVQMLVNMFYVL	-	-
594	YLRSTNWDTRIAAGQAVEAIVK	-	-

Table S2 The pharmacokinetic properties and physicochemical properties of 105 peptides and IPI were further investigated by ADME evaluation

Number	Molecule	Canonical SMILES	Formula	MW	#Heavy atoms	#Aromatic heavy atoms	Fraction Csp3	#Rotatable bonds	#H-bond acceptors	#H-bond donors	MR	TPSA	iLOGP	XLOGP3	WLOGP	MLOGP	Silicos-IT Log P	Consensus Log P	ESOL Log S	ESOL Solubility (mg/ml)	ESOL Solubility (mol/l)	ESOL Class	Ali Log S	Ali Solubility (mg/ml)	Ali Solubility (mol/l)	Ali Class	Silicos-IT LogSw	Silicos-IT Solubility (mg/ml)	Silicos-IT Solubility (mol/l)	Silicos-IT class	GI absorption	BBB permeant	Pgp substrate	CYP1A2 inhibitor	CYP2C19 inhibitor	CYP2C9 inhibitor	CYP2D6 inhibitor	CYP3A4 inhibitor	log Kp (cm/s)	Lipinski #violations	Ghose #violations	Veber #violations	Egan #violations	Muegge #violations	Bioavailability Score	PAINS #alerts	Brenk #alerts	Leadlikeness #violations	Synthetic Accessibility
1	FRPD	NC(=N)NCCC[C@@H](C(=O)N1CCC[C@H]1C(=O)N[C@H](C(=O)O)CC(=O)O)NC(=O)[C@H](Cc1ccccc1)N	C24H35N7O7	533.58	38	6	0.5	18	9	8	139.38	241.03	1.04	-5.06	-2.04	-1.36	-0.78	-1.64	1.11	6890	12.9	Highly soluble	0.64	2320	4.34	Highly soluble	-2.69	1.09	0.00205	Soluble	Low	No	No	No	No	No	No	No	-13.15	3	4	2	1	4	0.17	0	2	2	4.68
2	QPPK	NCCCC[C@@H](C(=O)O)NC(=O)[C@@H]1CCCN1C(=O)[C@@H]1CCCN1C(=O)[C@H](CCC(=O)N)N	C21H36N6O6	468.55	33	0	0.76	15	8	5	125.95	202.15	1.66	-5.55	-2.5	-1.82	-1	-1.84	1.74	25800	55.1	Highly soluble	1.96	43000	91.8	Highly soluble	-0.84	68.4	0.146	Soluble	Low	No	No	No	No	No	No	No	-13.1	1	1	2	1	2	0.55	0	0	2	4.39
3	PPG	NC(=N)NCCC[C@@H](C(=O)O)NC(=O)CNC(=O)[C@@H]1CC	C18	425	30	0	0.7	14	7	7	115	189	1.0	-4.	-3.	-1.	-1.3	-1.8	0.9	4170	9.8	Hig	0.6	1930	4.53	Highl	-1.7	8.45	0.0199	Solu	Low	No	No	No	No	No	No	No	-11	2	1	2	1	3	0.17	0	2	2	4.03

	R	CN1C(=O)[C@@H]1CCCN1	H31N7O5	.48			2				.01	.74	1	04	08	69	5	3	9			hly soluble	6			y soluble				ble									.76										
4	FDQ	OC(=O)C[C@@H](C(=O)N[C@H](C(=O)O)CCC(=O)N)NC(=O)[C@H](Cc1ccccc1)N	C18H24N4O7	408.41	29	6	0.39	14	8	6	99.45	201.91	-0.26	-4.04	-1.65	-1.19	-0.55	-1.54	0.94	3590	8.79	Highly soluble	0.4	1030	2.52	Highly soluble	-2.06	3.59	0.00879	Soluble	Low	No	Yes	No	No	No	No	No	-11.66	2	1	2	1	3	0.11	0	0	2	3.7
5	EAPF	OC(=O)CC[C@@H](C(=O)N[C@H](C(=O)N1CCC[C@H]1C(=O)N[C@H](C(=O)O)Cc1ccccc1)C)N	C22H30N4O7	462.5	33	6	0.5	14	8	5	120.66	179.13	1.92	-3.89	-0.89	-0.68	0.27	-0.66	0.53	1580	3.41	Highly soluble	0.72	2450	5.29	Highly soluble	-2.56	1.27	0.00275	Soluble	Low	No	No	No	No	No	No	No	-11.88	1	1	2	1	2	0.11	0	0	2	4.04
6	LKPP	NCCCC[C@@H](C(=O)N1CCC[C@H]1C(=O)N1CCC[C@H]1C(=O)O)NC(=O)[C@H](CC(C)C)N	C22H39N5O5	453.58	32	0	0.82	14	7	4	127.85	159.06	2.59	-3.09	-0.72	-0.4	0.49	-0.23	0.22	750	1.65	Highly solu	0.32	937	2.07	Highly soluble	-1.68	9.48	0.0209	Soluble	Low	No	Yes	No	No	No	No	No	-11.26	0	2	2	1	2	0.55	0	0	2	4.39

																						ble																											
7	LEGF	CC(C[C@@H](C(=O)N[C@H](C(=O)NCC(=O)N[C@H](C(=O)O)Cc1ccccc1)CCC(=O)O)N)C	C22H32N4O7	464.51	33	6	0.5	17	8	6	118.77	187.92	1.88	-2.25	-0.36	-0.29	1.01	0	-0.32	225	0.484	Very soluble	-1.16	31.9	0.0688	Very soluble	-3.64	0.106	0.000229	Soluble	Low	No	Yes	No	No	No	No	No	-10.73	2	0	2	1	4	0.11	0	0	2	3.98
8	FYPD	OC(=O)C[C@@H](C(=O)O)NC(=O)[C@@H]1CCCN1C(=O)[C@H](Cc1ccc(cc1)O)NC(=O)[C@H](Cc1ccccc1)N	C27H32N4O8	540.56	39	12	0.37	15	9	6	142.37	199.36	1.49	-1.5	-0.36	-0.35	0.9	0.04	-1.48	17.7	0.0328	Very soluble	-2.18	3.56	0.00659	Soluble	-4.03	0.0509	0.000942	Moderately soluble	Low	No	No	No	No	No	No	No	-10.66	3	3	2	1	2	0.11	0	0	2	4.48
9	FEY	OC(=O)CC[C@@H](C(=O)N[C@H](C(=O)O)Cc1ccc(cc1)O)NC(=O)[C@H](Cc1ccccc1)N	C23H27N3O7	457.48	33	12	0.3	14	8	6	118.24	179.05	1.47	-2.45	0.42	0.4	1.38	0.25	-0.48	152	0.333	Very soluble	-0.77	77.9	0.17	Very soluble	-4.36	0.0199	0.000436	Moderately soluble	Low	No	Yes	No	No	No	No	No	-10.83	1	0	2	1	3	0.11	0	0	2	3.81
10	PPQP	NC(=O)CC[C@@H](C(=O)N1CCC[C@H]1C(=O)O)NC(=O)[C@@H]1CCCN1C(=O)[C@@H]1CCCN1	C20H31N5O6	437.49	31	0	0.75	11	7	4	120.33	162.14	2.17	-3.68	-2.59	-1.29	-0.81	-1.24	0.49	1360	3.1	Highly solu	0.86	3190	7.29	Highly soluble	-0.85	62.1	0.142	Soluble	Low	No	No	No	No	No	No	No	-11.58	1	1	2	1	2	0.55	0	0	2	4.11

																						ble																											
11	WKP	NCCCC[C@@H](C(=O)N1CCC[C@H]1C(=O)O)NC(=O)[C@H](Cc1c[nH]c2c1cccc2)N	C22H31N5O4	429.51	31	9	0.5	12	6	5	120.84	154.54	1.97	-1.47	0.35	-0.1	1.59	0.47	-1	43	0.1	Very soluble	-1.27	23	0.0536	Very soluble	-4.12	0.0326	0.0000759	Moderately soluble	Low	No	Yes	No	No	No	No	No	-9.96	0	0	2	1	1	0.55	0	0	2	3.95
12	HPF	OC(=O)[C@H](Cc1ccccc1)NC(=O)[C@@H]1CCCN1C(=O)[C@H](Cc1cnc[nH]1)N	C20H25N5O4	399.44	29	11	0.4	10	6	4	108.49	141.41	1.88	-2.45	-0.3	-0.44	1.08	-0.05	-0.39	161	0.404	Very soluble	0.02	420	1.05	Highly soluble	-3.76	0.0694	0.000174	Soluble	Low	No	No	No	No	No	No	No	-10.48	0	0	1	1	1	0.55	0	0	2	3.83
13	WAH	O=C([C@@H](NC(=O)[C@H](Cc1c[nH]c2c1cccc2)N)C)N[C@H](C(=O)O)Cc1cnc[nH]1	C20H24N6O4	412.44	30	14	0.3	11	6	6	108.84	165.99	0.99	-2.82	0.08	-1.09	1.57	-0.26	-0.24	237	0.576	Very soluble	-0.11	320	0.775	Very soluble	-4.91	0.0051	0.0000124	Moderately soluble	Low	No	No	No	No	No	No	No	-10.82	1	0	2	1	3	0.55	0	0	2	3.83
14	MPFPK	CSCC[C@@H](C(=O)N1CCC[C@H]1C(=O)N[C@H](C(=O)N1CCC[C@H]1C(=O)N[C@H](C(=O)O)CCCCN)Cc1ccccc1)N	C30H46N6O6	618.79	43	6	0.63	20	8	5	172.54	213.46	3.27	-1.41	-0.29	-0.21	1.78	0.63	-1.57	16.6	0.0268	Very solubl	-2.57	1.66	0.00269	Soluble	-4.53	0.0182	0.0000294	Moderately solubl	Low	No	No	No	No	No	No	No	-11.08	2	3	2	1	3	0.17	0	0	2	5.49

			S																			e								e																			
15	LEF	CC(C[C@@H](C(=O)N[C@H](C(=O)N[C@H](C(=O)O)Cc1ccccc1)CCC(=O)O)N)C	C20H29N3O6	407.46	29	6	0.5	14	7	5	106.15	158.82	2.28	-2.18	0.52	0.48	1.34	0.49	-0.22	244	0.6	Very soluble	-0.62	96.8	0.238	Very soluble	-3.29	0.209	0.000513	Soluble	Low	No	Yes	No	No	No	No	No	-10.33	0	0	2	1	2	0.11	0	0	2	3.72
16	FLE	CC(C[C@@H](C(=O)N[C@H](C(=O)O)CCC(=O)O)NC(=O)[C@H](Cc1ccccc1)N)C	C20H29N3O6	407.46	29	6	0.5	14	7	5	106.15	158.82	1.86	-1.94	0.52	0.48	1.34	0.45	-0.37	173	0.423	Very soluble	-0.87	54.6	0.134	Very soluble	-3.29	0.209	0.000513	Soluble	Low	No	Yes	No	No	No	No	No	-10.16	0	0	2	1	1	0.11	0	0	2	3.69
17	FEL	CC(C[C@@H](C(=O)O)NC(=O)[C@@H](NC(=O)[C@H](Cc1ccccc1)N)CCC(=O)O)C	C20H29N3O6	407.46	29	6	0.5	14	7	5	106.15	158.82	1.73	-1.94	0.52	0.48	1.34	0.43	-0.37	173	0.423	Very soluble	-0.87	54.6	0.134	Very soluble	-3.29	0.209	0.000513	Soluble	Low	No	Yes	No	No	No	No	No	-10.16	0	0	2	1	1	0.11	0	0	2	3.67
18	LFE	CC(C[C@@H](C(=O)N[C@H](C(=O)N[C@H](C(=O)O)CCC(=O)O)Cc1ccccc1)N)C	C20H29N3O6	407.46	29	6	0.5	14	7	5	106.15	158.82	1.25	-1.56	0.52	0.48	1.34	0.41	-0.61	99.4	0.244	Very soluble	-1.27	22	0.054	Very soluble	-3.29	0.209	0.000513	Soluble	Low	No	Yes	No	No	No	No	No	-9.89	0	0	2	1	1	0.11	0	0	2	3.74
1	W	NC(=N)NCCC[C@@	C	4	3	9	0	1	7	9	1	2	0	-	-	-	0.	-	0	16	3.	H	-	12	0.	V	-	0.0	0.0	M	L	N	N	N	N	N	N	N	-	2	2	2	1	4	0.	0	2	2	4.

9	RQ	H](C(=O)N[C@H](C(=O)O)CCC(=O)N)NC(=O)[C@H](Cc1c[nH]c2c1cccc2)N	22H32N8O5	88.54	5		.41	7			28.25	42.3	.48	3.91	1.38	1.66	02	1.29	.53	40	36	ighlysoluble	0.58	8	262	erysoluble	4.08	403	000824	oderatelysoluble	ow	o	o	o	o	o	o	o	12.06						17				29
20	APAY	Oc1ccc(cc1)C[C@@H](C(=O)O)NC(=O)[C@@H](NC(=O)[C@@H]1CCCN1C(=O)[C@@H](N)C)C	C20H28N4O6	420.46	30	6	0.5	11	7	5	111.3	1162.06	1.81	-2.68	-1.03	-0.82	0.01	-0.54	-0.18	278	0.66	Verysoluble	-0.17	282	0.671	Verysoluble	-2.23	2.47	0.00587	Soluble	Low	No	No	No	No	No	No	No	-10.77	0	1	2	1	2	0.55	0	0	2	3.75
21	FTR	NC(=N)NCCC[C@@H](C(=O)O)NC(=O)[C@H]([C@H](O)C)NC(=O)[C@H](Cc1ccccc1)N	C19H30N6O5	422.48	30	6	0.47	15	7	8	109.84	203.65	0.74	-4.09	-1.75	-0.88	-0.5	-1.3	0.96	3850	9.11	Highlysoluble	0.42	1100	2.61	Highlysoluble	-2.73	0.792	0.00187	Soluble	Low	No	Yes	No	No	No	No	No	-11.78	2	1	2	1	3	0.17	0	2	2	4.05
22	WDL	CC(C[C@@H](C(=O)O)NC(=O)[C@@H](NC(=O)[C@H](Cc1c[nH]c2c1cccc2)N)CC(=O)O)C	C21H28N4O6	432.47	31	9	0.43	13	7	6	113.2	174.61	1.29	-1.85	0.61	-0.35	1.54	0.25	-0.71	83.8	0.194	Verysolubl	-1.3	21.8	0.0503	Verysoluble	-3.75	0.0773	0.00179	Soluble	Low	No	No	No	No	No	No	No	-10.25	1	0	2	1	2	0.11	0	0	2	3.96

																						e																											
23	WLD	CC(C[C@@H](C(=O)N[C@H](C(=O)O)CC(=O)O)NC(=O)[C@H](Cc1c[nH]c2c1cccc2)N)C	C21H28N4O6	432.47	31	9	0.43	13	7	6	113.2	174.61	0.24	-1.85	0.61	-0.35	1.54	0.04	-0.71	83.8	0.194	Very soluble	-1.3	21.8	0.0503	Very soluble	-3.75	0.0773	0.0000179	Soluble	Low	No	No	No	No	No	No	No	-10.25	1	0	2	1	2	0.11	0	0	2	3.94
24	LEW	CC(C[C@@H](C(=O)N[C@H](C(=O)N[C@H](C(=O)O)Cc1c[nH]c2c1cccc2)CCC(=O)O)N)C	C22H30N4O6	446.5	32	9	0.45	14	7	6	118.01	174.61	1.84	-1.65	1	-0.13	1.96	0.6	-0.85	62.6	0.14	Very soluble	-1.51	13.9	0.0312	Very soluble	-4.14	0.0323	0.0000724	Moderately soluble	Low	No	Yes	No	No	No	No	No	-10.2	1	0	2	1	2	0.11	0	0	2	4.03
25	WLE	CC(C[C@@H](C(=O)N[C@H](C(=O)O)CCC(=O)O)NC(=O)[C@H](Cc1c[nH]c2c1cccc2)N)C	C22H30N4O6	446.5	32	9	0.45	14	7	6	118.01	174.61	0.7	-1.5	1	-0.13	1.96	0.41	-0.95	50.4	0.113	Very soluble	-1.66	9.74	0.0218	Very soluble	-4.14	0.0323	0.0000724	Moderately soluble	Low	No	No	No	No	No	No	No	-10.09	1	0	2	1	2	0.11	0	0	2	4.01
26	FDF	OC(=O)C[C@@H](C(=O)N[C@H](C(=O)O)Cc1ccccc1)NC(=O)[C@H](Cc1ccccc1)N	C22H25N3O6	427.45	31	12	0.27	13	7	5	111.41	158.82	1.34	-2.48	0.33	0.69	1.43	0.26	-0.36	188	0.44	Very soluble	-0.31	208	0.486	Very soluble	-4.56	0.0117	0.0000275	Moderately soluble	Low	No	Yes	No	No	No	No	No	-10.67	0	0	2	1	2	0.11	0	0	2	3.78
2	R	NC(=N)NCCC[C@@	C	4	3	9	0	1	6	8	1	1	0	-	-	-	0.	-	0	78	1.	H	-	20	0.	V	-	0.0	0.0	M	L	N	N	N	N	N	N	N	-	2	1	2	1	3	0.	0	2	2	3.

7	AW	H](C(=O)N[C@H](C(=O)N[C@H](C(=O)O)Cc1c[nH]c2c1cccc2)C)N	20H29N7O4	31.49	1		.4	4			15.72	99.21	.1	3.28	0.62	0.93	44	0.86	.26	6	82	ighlysoluble	0.33	1	467	erysoluble	4.13	319	00074	oderatelysoluble	ow	o	o	o	o	o	o	o	11.26						17				99
28	QPY	NC(=O)CC[C@@H](C(=O)N1CCC[C@H]1C(=O)N[C@H](C(=O)O)Cc1ccc(cc1)O)N	C19H26N4O6	406.43	29	6	0.47	11	7	5	106.4	176.05	1.03	-4.13	-1.29	-1.05	-0.3	-1.15	0.81	2650	6.53	Highly soluble	1.04	4430	10.9	Highly soluble	-1.82	6.17	0.0152	Soluble	Low	No	No	No	No	No	No	No	-11.71	0	1	2	1	2	0.55	0	0	2	3.55
29	YPSP	OC[C@@H](C(=O)N1CCC[C@H]1C(=O)O)NC(=O)[C@@H]1CCCN1C(=O)[C@H](Cc1ccc(cc1)O)N	C22H30N4O7	462.5	33	6	0.55	11	8	5	123.97	173.5	1.28	-3.28	-1.96	-1.14	-0.5	-1.12	-0.05	413	0.892	Very soluble	0.21	748	1.62	Highly soluble	-1.36	20.2	0.0438	Soluble	Low	No	No	No	No	No	No	No	-11.45	1	1	2	1	2	0.55	0	0	2	4
30	FNQ	NC(=O)C[C@@H](C(=O)N[C@H](C(=O)O)CCC(=O)N)NC(=O)[C@H](Cc1ccccc1)N	C18H25N5O6	407.42	29	6	0.39	14	7	6	100.58	207.7	0.15	-4.69	-2.25	-1.59	-0.79	-1.83	1.36	9320	22.9	Highlysolu	0.95	3660	8.99	Highly soluble	-2.27	2.17	0.00533	Soluble	Low	No	Yes	No	No	No	No	No	-12.12	2	1	2	1	3	0.17	0	0	2	3.65

																						ble																											
31	LPAPIVR	CC[C@@H]([C@@H](C(=O)N[C@H](C(=O)N[C@H](C(=O)O)CCCNC(=N)N)C(C)C)NC(=O)[C@@H]1CCCN1C(=O)[C@@H](NC(=O)[C@@H]1CCCN1C(=O)[C@H](CC(C)C)N)C)C	C36H64N10O8	764.96	54	0	0.78	27	10	9	210.55	282.24	4.09	-1.12	-1.41	-1.49	0.97	0.21	-2.1	6.14	0.00803	Soluble	-4.32	0.037	0.0000483	Moderately soluble	-3.59	0.197	0.000257	Soluble	Low	No	Yes	No	No	No	No	No	-11.76	3	4	2	1	4	0.17	0	2	2	6.94
32	SPFQ	OC[C@@H](C(=O)N1CCC[C@H]1C(=O)N[C@H](C(=O)N[C@@H](C(=O)O)CCC(=O)N)Cc1ccccc1)N	C22H31N5O7	477.51	34	6	0.5	15	8	6	122.96	205.15	1.19	-5.51	-2.52	-1.84	-0.56	-1.85	1.53	16200	33.9	Highly soluble	1.86	34500	72.2	Highly soluble	-2.2	2.99	0.000627	Soluble	Low	No	No	No	No	No	No	No	-13.12	2	1	2	1	3	0.17	0	0	2	4.22
33	FHVM	CSCC[C@@H](C(=O)O)NC(=O)[C@H](C(C)C)NC(=O)[C@@H](NC(=O)[C@H](Cc1ccccc1)N)Cc1[nH]cnc1	C25H36N6O5S	532.66	37	11	0.48	18	7	6	141.22	204.6	2.59	-2.19	0.47	-0.56	2.46	0.55	-0.79	85.4	0.16	Very soluble	-1.58	14.2	0.0266	Very soluble	-5.69	0.0011	0.0000206	Moderately soluble	Low	No	Yes	No	No	No	No	No	-11.1	3	3	2	1	4	0.17	0	0	2	4.94
34	SPPL	OC[C@@H](C(=O)N1CCC[C@H]1C(=O)N1CCC[C@H]1C(=O)N[C@H](C(=O)O)CC(C)C)N	C19H32N4	412.48	29	0	0.79	11	7	4	111.88	153.27	1.89	-3.88	-1.86	-1.07	-0.5	-1.08	0.77	2450	5.93	Highly so	1.26	7440	18	Highly soluble	-0.29	212	0.514	Soluble	Low	No	No	No	No	No	No	No	-11.57	0	1	2	1	2	0.55	0	0	2	4.04

			O6																			luble																											
35	LWK	NCCCC[C@@H](C(=O)O)NC(=O)[C@H](Cc1c[nH]c2c1cccc2)NC(=O)[C@H](CC(C)C)N	C23H35N5O4	445.56	32	9	0.52	15	6	6	123.75	163.33	1.46	-1.22	1.27	0.12	2.56	0.84	-1.05	39.5	0.0887	Very soluble	-1.72	8.59	0.0193	Very soluble	-5.22	0.0027	0.000000607	Moderately soluble	Low	No	Yes	No	No	No	No	No	-9.88	1	0	2	1	2	0.55	0	0	2	4.19
36	LRPL	NC(=N)NCCC[C@@H](C(=O)N1CCC[C@H]1C(=O)N[C@H](C(=O)O)CC(C)C)NC(=O)[C@H](CC(C)C)N	C23H43N7O5	497.63	35	0	0.78	18	7	7	137.15	203.73	2.75	-3.14	-0.67	-0.57	0.45	-0.24	0.24	867	1.74	Highly soluble	-0.57	134	0.268	Very soluble	-2.49	1.63	0.00327	Soluble	Low	No	No	No	No	No	No	No	-11.56	2	4	2	1	4	0.17	0	2	2	4.85
37	PAAF	O=C([C@@H](NC(=O)[C@@H](NC(=O)[C@@H]1CCCN1)C)C)N[C@H](C(=O)O)Cc1ccccc1	C20H28N4O5	404.46	29	6	0.5	12	6	5	109.28	136.63	2.27	-2.58	-0.82	-0.32	0.98	-0.09	-0.08	334	0.825	Very soluble	0.26	731	1.81	Highly soluble	-3.95	0.0458	0.000113	Soluble	Low	No	No	No	No	No	No	No	-10.6	0	1	1	1	1	0.55	0	0	2	3.7
38	LELF	CC(C[C@@H](C(=O)N[C@H](C(=O)O)Cc1ccccc1)NC(=O)[C@@H](NC(=O)[C@H](CC(C)C)N)CCC(=O)O)C	C26H40	520.62	37	6	0.58	19	8	6	138	187.92	1.95	-1.59	1.05	0.55	2.37	0.87	-0.93	60.9	0.117	Very so	-1.85	7.4	0.0142	Very solubl	-4.45	0.0185	0.00000356	Moderatel	Low	No	Yes	No	No	No	No	No	-10.6	3	3	2	1	3	0.11	0	0	2	4.62

			N4O7																			luble				e				y soluble																			
39	FELL	CC(C[C@@H](C(=O)N[C@H](C(=O)O)CC(C)C)NC(=O)[C@@H](NC(=O)[C@H](Cc1ccccc1)N)CCC(=O)O)C	C26H40N4O7	520.62	37	6	0.58	19	8	6	138	187.92	1.24	-1.59	1.05	0.55	2.37	0.72	-0.93	60.9	0.117	Very soluble	-1.85	7.4	0.0142	Very soluble	-4.45	0.0185	0.000356	Moderately soluble	Low	No	Yes	No	No	No	No	No	-10.6	3	3	2	1	3	0.11	0	0	2	4.57
40	FRY	NC(=N)NCCC[C@@H](C(=O)N[C@H](C(=O)O)Cc1ccc(cc1)O)NC(=O)[C@H](Cc1ccccc1)N	C24H32N6O5	484.55	35	12	0.33	16	7	8	130.38	203.65	1.14	-3.16	-0.18	0.26	0.93	-0.2	-0.05	431	0.889	Very soluble	-0.55	137	0.283	Very soluble	-5.15	0.00343	0.0000708	Moderately soluble	Low	No	Yes	No	No	No	No	No	-11.5	2	2	2	1	4	0.17	0	2	2	4.13
41	FDVW	OC(=O)C[C@@H](C(=O)N[C@H](C(=O)N[C@H](C(=O)O)Cc1c[nH]c2c1cccc2)C(C)C)NC(=O)[C@H](Cc1ccccc1)N	C29H35N5O7	565.62	41	15	0.34	17	8	7	150.31	203.71	0.8	-1.2	0.95	-0.09	2.54	0.6	-1.74	10.3	0.0182	Very soluble	-2.58	1.47	0.00261	Soluble	-6.17	0.000387	0.00000684	Poorly soluble	Low	No	No	No	No	No	No	No	-10.6	3	3	2	1	3	0.11	0	0	2	4.81
42	FPPQ	NC(=O)CC[C@@H](C(=O)O)NC(=O)[C@@H]1CCCN1C(=O)[C@@H]1CCCN1C(=O)[C@H](Cc1ccccc1)N	C24H33N5	487.55	35	6	0.54	13	7	4	133.31	176.13	1.83	-2.72	-1.39	-0.66	0.1	-0.57	-0.42	186	0.382	Very solu	-0.43	182	0.374	Very soluble	-2.48	1.63	0.00333	Soluble	Low	No	No	No	No	No	No	No	-11.21	1	3	2	1	2	0.55	0	0	2	4.22

			O6																			ble																											
43	LLR	CC(C[C@@H](C(=O)N[C@H](C(=O)O)CCCNC(=N)N)NC(=O)[C@H](CC(C)C)N)C	C18H36N6O4	400.52	28	0	0.78	16	6	7	108.22	183.42	1.45	-3.08	-0.28	-0.1	0.38	-0.32	0.67	1890	4.71	Highly soluble	-0.21	249	0.621	Very soluble	-2.42	1.53	0.00381	Soluble	Low	No	Yes	No	No	No	No	No	-10.93	1	0	2	1	4	0.55	0	2	2	4.15
44	LRL	NC(=N)NCCC[C@@H](C(=O)N[C@H](C(=O)O)CC(C)C)NC(=O)[C@H](CC(C)C)N	C18H36N6O4	400.52	28	0	0.78	16	6	7	108.22	183.42	1.27	-3.08	-0.28	-0.1	0.38	-0.36	0.67	1890	4.71	Highly soluble	-0.21	249	0.621	Very soluble	-2.42	1.53	0.00381	Soluble	Low	No	Yes	No	No	No	No	No	-10.93	1	0	2	1	4	0.55	0	2	2	4.14
45	TPPL	CC(C[C@@H](C(=O)O)NC(=O)[C@@H]1CCCN1C(=O)[C@@H]1CCCN1C(=O)[C@H]([C@H](O)C)N)C	C20H34N4O6	426.51	30	0	0.8	11	7	4	116.69	153.27	2.48	-3.45	-1.47	0.84	-0.27	-0.71	0.42	1110	2.6	Highly soluble	0.81	2750	6.46	Highly soluble	-0.31	210	0.493	Soluble	Low	No	No	No	No	No	No	No	-11.35	0	1	2	1	2	0.55	0	0	2	4.28
46	WAQ	NC(=O)CC[C@@H](C(=O)O)NC(=O)[C@@H](NC(=O)[C@H](Cc1c[nH]c2c1cccc2)N	C19H	403.	29	9	0.37	12	6	6	104.	180.	1.3	-3.4	-0.6	-1.2	0.65	-0.66	0.36	931	2.31	High	0.19	623	1.54	Highly	-3.56	0.112	0.000279	Solubl	Low	No	No	No	No	No	No	No	-11.	1	1	2	1	3	0.55	0	0	2	3.69

		)C	25N5O5	43							72	4			2							lysoluble				soluble				e									17										
47	PLPP	CC(C[C@@H](C(=O)N1CCC[C@H]1C(=O)N1CCC[C@H]1C(=O)O)NC(=O)[C@@H]1CCCN1)C	C21H34N4O5	422.52	30	0	0.81	10	6	3	122.23	119.05	2.36	-1.2	-0.81	0.15	0.69	0.24	-1.04	38.2	0.0904	Very soluble	-0.81	66	0.156	Very soluble	-1.69	8.63	0.0204	Soluble	High	No	Yes	No	No	No	No	No	-9.73	0	1	0	0	0	0.55	0	0	2	4.07
48	PFSP	OC[C@@H](C(=O)N1CCC[C@H]1C(=O)O)NC(=O)[C@H](Cc1ccccc1)NC(=O)[C@@H]1CCCN1	C22H30N4O6	446.5	32	6	0.55	12	7	5	121.95	148.07	2.58	-2.28	-1.74	-0.65	0.47	-0.32	-0.52	135	0.303	Very soluble	-0.29	227	0.507	Very soluble	-3.08	0.375	0.000839	Soluble	Low	No	No	No	No	No	No	No	-10.64	0	1	2	1	1	0.55	0	0	2	4.01
49	WTN	NC(=O)C[C@@H](C(=O)O)NC(=O)[C@H]([C@H](O)C)NC(=O)[C@H](Cc1c[nH]c2c1cccc2)N	C19H25N5O6	419.43	30	9	0.37	12	7	7	105.89	200.63	0.17	-4.37	-1.65	-1.97	-0.1	-1.59	0.88	3200	7.63	Highly soluble	0.77	2470	5.89	Highly soluble	-2.61	1.03	0.00245	Soluble	Low	No	No	No	No	No	No	No	-11.96	2	1	2	1	3	0.17	0	0	2	3.95
50	WF	NCCCC[C@@H](C(=O)O)NC(=O)[C@@H	C2	47	35	15	0.	15	6	6	13	16	2.	-1	1.	0.	3.0	1.2	-1	21	0.04	Ve	-1	9.69	0.02	Ver	-6.	0.000	0.000	Po	Lo	No	Ye	No	No	No	No	No	-1	1	1	2	1	2	0.55	0	0	2	4.21

	K	](NC(=O)[C@H](Cc1c[nH]c2c1cccc2)N)Cc1ccccc1	6H33N5O4	9.57			35				3.82	3.33	65	.24	46	52	3	9	.36		37	rysoluble	.69		02	ysoluble	88	0638	000133	orlysoluble	w		s						0.11										
51	LHW	CC(C[C@@H](C(=O)N[C@H](C(=O)N[C@@H](C(=O)O)Cc1c[nH]c2c1cccc2)Cc1[nH]cnc1)N)C	C23H30N6O4	454.52	33	14	0.39	13	6	6	123.26	165.99	1.05	-1.31	1.1	-0.43	2.62	0.6	-1.29	23.4	0.0514	Verysoluble	-1.68	9.55	0.021	Verysoluble	-5.71	0.00089	0.0000196	Moderatelysoluble	Low	No	No	No	No	No	No	No	-10	1	0	2	1	2	0.55	0	0	2	4.21
52	WLH	CC(C[C@@H](C(=O)N[C@H](C(=O)O)Cc1cnc[nH]1)NC(=O)[C@H](Cc1c[nH]c2c1cccc2)N)C	C23H30N6O4	454.52	33	14	0.39	13	6	6	123.26	165.99	0.14	-1.22	1.1	-0.43	2.62	0.44	-1.35	20.5	0.0451	Verysoluble	-1.77	7.7	0.0169	Verysoluble	-5.71	0.00089	0.0000196	Moderatelysoluble	Low	No	No	No	No	No	No	No	-9.94	1	0	2	1	2	0.55	0	0	2	4.17
53	FEFL	CC(C[C@@H](C(=O)O)NC(=O)[C@@H](NC(=O)[C@@H](NC(=O)[C@H](Cc1ccccc1)N)CCC(=O)O)Cc1ccccc1)C	C29H38N4O7	554.63	40	12	0.41	19	8	6	148.06	187.92	2.26	-0.23	1.25	0.93	2.82	1.4	-2.1	4.39	0.00791	Soluble	-3.26	0.306	0.000551	Soluble	-6.1	0.000439	0.00000791	Poorlysoluble	Low	No	Yes	No	No	No	No	No	-9.85	3	3	2	1	3	0.11	0	0	2	4.66
54	YPY	Oc1ccc(cc1)C[C@@H](C(=O)O)NC(=O)[C@@H]1CCCN1C(=O)[C@H](Cc1ccc(cc1)O)N	C23H2	441.4	32	12	0.35	10	7	5	120.3	153.1	1.35	-1.05	0.39	0.31	1.21	0.44	-1.53	12.9	0.0293	Verys	-1.68	9.25	0.021	Verysolu	-3.73	0.0815	0.00185	Soluble	Low	No	Yes	No	No	No	No	No	-9.74	0	0	1	1	1	0.55	0	0	2	3.66

			7N3O6	8							9	9										oluble				ble																							
55	FAVP	O=C([C@@H](NC(=O)[C@H](Cc1ccccc1)N)C)N[C@H](C(=O)N1CCC[C@H]1C(=O)O)C(C)C	C22H32N4O5	432.51	31	6	0.55	12	6	4	118.89	141.83	2.22	-0.9	-0.1	0.12	1.11	0.49	-1.31	21.4	0.0495	Very soluble	-1.6	11	0.0254	Very soluble	-3.23	0.255	0.000589	Soluble	Low	No	Yes	No	No	No	No	No	-9.58	0	0	2	1	0	0.55	0	0	2	3.9
56	VAPF	OC(=O)[C@H](Cc1ccccc1)NC(=O)[C@@H]1CCCN1C(=O)[C@@H](NC(=O)[C@H](C(C)C)N)C	C22H32N4O5	432.51	31	6	0.55	12	6	4	118.89	141.83	2.7	-2.41	-0.1	0.12	1.11	0.28	-0.35	191	0.442	Very soluble	-0.03	405	0.936	Very soluble	-3.23	0.255	0.000589	Soluble	Low	No	Yes	No	No	No	No	No	-10.65	0	0	2	1	1	0.55	0	0	2	3.94
57	TPW	OC(=O)[C@H](Cc1c[nH]c2c1cccc2)NC(=O)[C@@H]1CCCN1C(=O)[C@H]([C@H](O)C)N	C20H26N4O5	402.44	29	9	0.45	9	6	5	109.68	148.75	1.87	-2.45	-0.4	-0.54	0.84	-0.13	-0.43	150	0.374	Very soluble	-0.13	297	0.737	Very soluble	-2.75	0.724	0.0018	Soluble	Low	No	No	No	No	No	No	No	-10.49	0	1	1	1	1	0.55	0	0	2	3.85
58	VPPF	OC(=O)[C@H](Cc1ccccc1)NC(=O)[C@@H]1CCCN1C(=O)[C@@H]1CCCN1C(=O)[C@H](C(C)C)N	C24H34N	458.55	33	6	0.58	11	6	3	130.4	133.04	2.86	-0.6	0	0.55	1.2	0.8	-1.71	8.87	0.0193	Very sol	-1.72	8.69	0.0189	Very soluble	-2.93	0.538	0.00117	Soluble	High	No	Yes	No	No	No	No	No	-9.52	0	1	1	1	0	0.55	0	0	2	4.09

			4O5																			uble																											
59	SPLL	OC[C@@H](C(=O)N1CCC[C@H]1C(=O)N[C@H](C(=O)N[C@H](C(=O)O)CC(C)C)CC(C)C)N	C20H36N4O6	428.52	30	0	0.8	14	7	5	114.8	162.06	2.3	-3.29	-0.94	-0.84	0.46	-0.46	0.5	1350	3.16	Highly soluble	0.46	1230	2.88	Highly soluble	-1.39	17.6	0.041	Soluble	Low	No	Yes	No	No	No	No	No	-11.25	0	1	2	1	2	0.55	0	0	2	4.24
60	FYA	O=C([C@H](Cc1ccc(cc1)O)NC(=O)[C@H](Cc1ccccc1)N)N[C@H](C(=O)O)C	C21H25N3O5	399.44	29	12	0.29	11	6	5	106.86	141.75	1.39	-1.93	0.58	0.78	1.58	0.48	-0.68	83.3	0.209	Very soluble	-0.53	119	0.298	Very soluble	-4.62	0.00958	0.000024	Moderately soluble	Low	No	Yes	No	No	No	No	No	-10.11	0	0	2	1	0	0.55	0	0	2	3.43
61	FLQ	CC(C[C@@H](C(=O)N[C@H](C(=O)O)CCC(=O)N)NC(=O)[C@H](Cc1ccccc1)N)C	C20H30N4O5	406.48	29	6	0.5	14	6	5	107.29	164.61	1.25	-2.59	-0.08	0.07	1.11	-0.05	0.04	448	1.1	Highly soluble	-0.32	194	0.478	Very soluble	-3.51	0.126	0.000311	Soluble	Low	No	Yes	No	No	No	No	No	-10.62	0	0	2	1	2	0.55	0	0	2	3.71
62	FALT	CC(C[C@@H](C(=O)N[C@H](C(=O)O)[C@H](O)C)NC(=O)[C@@H](NC(=O)[C@H](Cc1ccccc1)N)C)C	C22H3	450.5	32	6	0.55	15	7	6	118.1	170.8	1.9	-2.28	-0.46	-0.26	1.1	0	-0.35	203	0.451	Verys	-0.77	76	0.169	Very solu	-3.36	0.195	0.000434	Soluble	Low	No	Yes	No	No	No	No	No	-10.6	1	1	2	1	3	0.55	0	0	2	4.17

			4N4O6	3							6	5										oluble				ble													7										
63	LATF	CC(C[C@@H](C(=O)N[C@H](C(=O)N[C@H](C(=O)N[C@H](C(=O)O)Cc1ccccc1)[C@H](O)C)C)N)C	C22H34N4O6	450.53	32	6	0.55	15	7	6	118.16	170.85	1.59	-2.94	-0.46	-0.26	1.1	-0.19	0.07	530	1.18	Highly soluble	-0.09	368	0.816	Very soluble	-3.36	0.195	0.0000434	Soluble	Low	No	Yes	No	No	No	No	No	-11.14	1	1	2	1	3	0.55	0	0	2	4.23
64	LPPL	CC(C[C@@H](C(=O)O)NC(=O)[C@@H]1CCCN1C(=O)[C@@H]1CCCN1C(=O)[C@H](CC(C)C)N)C	C22H38N4O5	438.56	31	0	0.82	12	6	3	125.14	133.04	2.72	-1.51	0.2	0.37	1.15	0.58	-0.82	67	0.153	Very soluble	-0.78	73.1	0.167	Very soluble	-1.66	9.55	0.0218	Soluble	High	No	Yes	No	No	No	No	No	-10.05	0	0	1	1	0	0.55	0	0	2	4.24
65	YPF	Oc1ccc(cc1)C[C@@H](C(=O)N1CCC[C@H]1C(=O)N[C@H](C(=O)O)Cc1ccccc1)N	C23H27N3O5	425.48	31	12	0.35	10	6	4	118.37	132.96	1.91	-0.69	0.68	0.82	1.68	0.88	-1.67	9.1	0.0214	Very soluble	-1.63	10	0.0236	Very soluble	-4.32	0.0202	0.0000474	Moderately soluble	High	No	Yes	No	No	No	No	No	-9.39	0	0	0	1	0	0.55	0	0	2	3.62
66	WAY	Oc1ccc(cc1)C[C@@H](C(=O)O)NC(=O)[C@@H](NC(=O)[C@H](Cc1c[nH]c2c1cccc2)N)C	C23H2	438.4	32	15	0.26	11	6	6	118.7	157.5	1.58	-1.38	1.06	0.17	2.16	0.72	-1.31	21.5	0.049	Verys	-1.43	16.4	0.0374	Very solu	-5.47	0.00149	0.00000339	Moderat	Low	No	Yes	No	No	No	No	No	-9.95	1	0	2	1	2	0.55	0	0	2	3.79

			6N4O5	8							14											oluble				ble				elysoluble																			
67	WYA	Oc1ccc(cc1)C[C@@H](C(=O)N[C@H](C(=O)O)C)NC(=O)[C@H](Cc1c[nH]c2c1cccc2)N	C23H26N4O5	438.48	32	15	0.26	11	6	6	118.71	157.54	1.18	-1.36	1.06	0.17	2.16	0.64	-1.32	20.9	0.0476	Very soluble	-1.45	15.6	0.0356	Very soluble	-5.47	0.00149	0.0000339	Moderately soluble	Low	No	No	No	No	No	No	No	-9.94	1	0	2	1	2	0.55	0	0	2	3.79
68	PALF	CC(C[C@@H](C(=O)N[C@H](C(=O)O)Cc1ccccc1)NC(=O)[C@@H](NC(=O)[C@@H]1CCCN1)C)C	C23H34N4O5	446.54	32	6	0.57	14	6	5	123.7	136.63	1.84	-1.26	0.21	0.33	2.04	0.63	-1.03	41.7	0.0934	Very soluble	-1.11	34.4	0.0771	Very soluble	-4.75	0.008	0.0000179	Moderately soluble	High	No	Yes	No	No	No	No	No	-9.92	0	0	1	1	0	0.55	0	0	2	4.05
69	LPAF	CC(C[C@@H](C(=O)N1CCC[C@H]1C(=O)N[C@H](C(=O)N[C@H](C(=O)O)Cc1ccccc1)C)N)C	C23H34N4O5	446.54	32	6	0.57	13	6	4	123.7	141.83	2.22	-1.97	0.29	0.33	1.53	0.48	-0.65	100	0.225	Very soluble	-0.49	146	0.327	Very soluble	-3.62	0.107	0.000239	Soluble	Low	No	Yes	No	No	No	No	No	-10.42	0	0	2	1	0	0.55	0	0	2	4.05
70	LRLF	CC(C[C@@H](C(=O)N[C@H](C(=O)O)Cc1ccccc1)NC(=O)[C@@H](NC(=O)[C@H](CC(C)C)N)CCCNC(=N)N)C	C27H45N	547.69	39	6	0.59	21	7	8	150.13	212.52	1.34	-1.9	0.45	0.4	1.94	0.45	-0.77	93.8	0.171	Verysol	-2.04	4.97	0.00907	Soluble	-5.23	0.0032	0.0000583	Moderately	Low	No	Yes	No	No	No	No	No	-10.99	3	3	2	1	3	0.17	0	2	2	4.97

			7O5																			uble								soluble																				
71	LQW	CC(C[C@@H](C(=O)N[C@H](C(=O)N[C@H](C(=O)O)Cc1c[nH]c2c1cccc2)CCC(=O)N)N)C	C22H31N5O5	445.51	32	9	0.45	14	6	6	119.15	180.4	2.91	-2.3	0.4	-0.53	1.72	0.44	-0.44	163	0.365	Very soluble	-0.95	49.7	0.111	Very soluble	-4.36	0.0196	0.000044	Moderately soluble	Low	No	Yes	No	No	No	No	No	-10.65	1	0	2	1	3	0.55	0	0	2	4.05
72	WQL	CC(C[C@@H](C(=O)O)NC(=O)[C@@H](NC(=O)[C@H](Cc1c[nH]c2c1cccc2)N)CCC(=O)N)C	C22H31N5O5	445.51	32	9	0.45	14	6	6	119.15	180.4	1.68	-2.15	0.4	-0.53	1.72	0.22	-0.53	131	0.294	Very soluble	-1.11	34.7	0.0779	Very soluble	-4.36	0.0196	0.000044	Moderately soluble	Low	No	Yes	No	No	No	No	No	-10.54	1	0	2	1	3	0.55	0	0	2	4.02
73	WLQ	CC(C[C@@H](C(=O)N[C@H](C(=O)O)CCC(=O)N)NC(=O)[C@H](Cc1c[nH]c2c1cccc2)N)C	C22H31N5O5	445.51	32	9	0.45	14	6	6	119.15	180.4	0.97	-2.15	0.4	-0.53	1.72	0.08	-0.53	131	0.294	Very soluble	-1.11	34.7	0.0779	Very soluble	-4.36	0.0196	0.000044	Moderately soluble	Low	No	No	No	No	No	No	No	-10.54	1	0	2	1	3	0.55	0	0	2	4.04
74	FQF	NC(=O)CC[C@@H](C(=O)N[C@H](C(=O)O)Cc1ccccc1)NC(=O)[C@H](Cc1ccccc1)N	C23H28N4O	440.49	32	12	0.3	14	6	5	117.35	164.61	1.41	-2.77	0.12	0.5	1.6	0.17	-0.18	291	0.662	Very solub	-0.13	324	0.735	Very soluble	-5.17	0.0298	0.00000678	Moderately solu	Low	No	Yes	No	No	No	No	No	-10.95	0	0	2	1	2	0.55	0	0	2	3.78

			5																			le								ble																			
75	FVY	CC([C@@H](C(=O)N[C@H](C(=O)O)Cc1ccc(cc1)O)NC(=O)[C@H](Cc1ccccc1)N)C	C23H29N3O5	427.49	31	12	0.35	12	6	5	116.47	141.75	1.32	-1.37	1.21	1.21	2.23	0.92	-1.12	32.3	0.0755	Very soluble	-1.11	33.5	0.0783	Very soluble	-5.03	0.00399	0.0000933	Moderately soluble	High	No	Yes	No	No	No	No	No	-9.88	0	0	2	1	0	0.55	0	0	2	3.67
76	WSL	OC[C@@H](C(=O)N[C@H](C(=O)O)CC(C)C)NC(=O)[C@H](Cc1c[nH]c2c1cccc2)N	C20H28N4O5	404.46	29	9	0.45	12	6	6	107.79	157.54	1.67	-2.03	0.13	-0.54	1.55	0.16	-0.51	126	0.312	Very soluble	-0.75	71.4	0.177	Very soluble	-3.83	0.0605	0.00015	Soluble	Low	No	Yes	No	No	No	No	No	-10.21	1	0	2	1	3	0.55	0	0	2	3.75
77	LSW	OC[C@@H](C(=O)N[C@H](C(=O)O)Cc1c[nH]c2c1cccc2)NC(=O)[C@H](CC(C)C)N	C20H28N4O5	404.46	29	9	0.45	12	6	6	107.79	157.54	1.77	-2.12	0.13	-0.54	1.55	0.16	-0.45	144	0.355	Very soluble	-0.66	88.6	0.219	Very soluble	-3.83	0.0605	0.00015	Soluble	Low	No	Yes	No	No	No	No	No	-10.27	1	0	2	1	3	0.55	0	0	2	3.77
78	LTW	CC(C[C@@H](C(=O)N[C@H](C(=O)N[C@H](C(=O)O)Cc1c[nH]c2c1cccc2)[C@H](O)C)N)C	C21H30N4O5	418.49	30	9	0.48	12	6	6	112.59	157.54	2.24	-1.69	0.52	-0.31	1.8	0.51	-0.8	66.3	0.159	Very soluble	-1.11	32.8	0.0784	Very soluble	-3.84	0.0599	0.000143	Soluble	Low	No	Yes	No	No	No	No	No	-10.05	1	0	2	1	2	0.55	0	0	2	4.03

79	WTL	CC(C[C@@H](C(=O)O)NC(=O)[C@H]([C@H](O)C)NC(=O)[C@H](Cc1c[nH]c2c1cccc2)N)C	C21H30N4O5	418.49	30	9	0.48	12	6	6	112.59	157.54	1.27	-1.6	0.52	-0.31	1.8	0.33	-0.86	58.2	0.139	Very soluble	-1.2	26.5	0.0632	Very soluble	-3.84	0.0599	0.000143	Soluble	Low	No	Yes	No	No	No	No	No	-9.99	1	0	2	1	2	0.55	0	0	2	4.01
80	LYL	Oc1ccc(cc1)C[C@@H](C(=O)N[C@H](C(=O)O)CC(C)C)NC(=O)[C@H](CC(C)C)N	C21H33N3O5	407.5	29	6	0.57	13	6	5	111.21	141.75	2.06	-1.34	1.41	1.01	2.15	1.06	-0.82	62	0.152	Very soluble	-1.14	29.7	0.0729	Very soluble	-3.76	0.0708	0.000174	Soluble	High	No	Yes	No	No	No	No	No	-9.74	0	0	2	1	0	0.55	0	0	2	3.74
81	WVY	Oc1ccc(cc1)C[C@@H](C(=O)O)NC(=O)[C@H](C(C)C)NC(=O)[C@H](Cc1c[nH]c2c1cccc2)N	C25H30N4O5	466.53	34	15	0.32	12	6	6	128.33	157.54	2.42	-0.42	1.7	0.58	2.81	1.42	-2	4.64	0.00995	Soluble	-2.42	1.76	0.00377	Soluble	-5.88	0.000618	0.0000132	Moderately soluble	Low	No	Yes	No	No	No	No	No	-9.44	1	0	2	1	2	0.55	0	0	2	4.02
82	FSW	OC[C@@H](C(=O)N[C@H](C(=O)O)Cc1c[nH]c2c1cccc2)NC(=O)[C@H](Cc1ccccc1)N	C23H26N4O5	438.48	32	15	0.26	12	6	6	117.85	157.54	2.09	-2.09	0.33	-0.1	2.03	0.45	-0.8	70	0.16	Very soluble	-0.69	89.4	0.204	Very soluble	-5.49	0.0143	0.0000325	Moderately soluble	Low	No	No	No	No	No	No	No	-10.46	1	0	2	1	3	0.55	0	0	2	3.83
83	WS	OC[C@@H](C(=O)N[C@H](C(=O)O)Cc1c	C2	43	32	15	0.	12	6	6	11	15	0.	-2	0.	-0	2.0	0.1	-0	70	0.16	Ve	-0	89.4	0.20	Ver	-5.	0.001	0.000	Mo	Lo	No	No	No	No	No	No	No	-1	1	0	2	1	3	0.55	0	0	2	3.83

	F	cccc1)NC(=O)[C@H](Cc1c[nH]c2c1cccc2)N	3H26N4O5	8.48			26				7.85	7.54	34	.09	33	.1	3		.8			rysoluble	.69		4	ysoluble	49	43	00325	derately soluble	w								0.46										
84	WLGL	CC(C[C@@H](C(=O)NCC(=O)N[C@H](C(=O)O)CC(C)C)NC(=O)[C@H](Cc1c[nH]c2c1cccc2)N)C	C25H37N5O5	487.59	35	9	0.52	16	6	6	133.66	166.41	1.84	0.06	1.3	0.1	2.87	1.23	-2.04	4.5	0.00922	Soluble	-3.11	0.38	0.00078	Soluble	-5.55	0.00139	0.0000284	Moderately soluble	Low	No	Yes	No	No	No	No	No	-9.23	1	3	2	1	3	0.55	0	0	2	4.29
85	WFT	N[C@H](C(=O)N[C@H](C(=O)N[C@H](C(=O)O)[C@H](O)C)Cc1ccccc1)Cc1c[nH]c2c1cccc2	C24H28N4O5	452.5	33	15	0.29	12	6	6	122.66	157.54	2.06	-1.62	0.72	0.11	2.27	0.71	-1.17	30.6	0.0677	Very soluble	-1.18	30	0.0663	Very soluble	-5.5	0.00141	0.0000313	Moderately soluble	Low	No	Yes	No	No	No	No	No	-10.21	1	0	2	1	2	0.55	0	0	2	4.08
86	LLPL	CC(C[C@@H](C(=O)N[C@H](C(=O)N1CCC[C@H]1C(=O)N[C@H](C(=O)O)CC(C)C)CC(C)C)N)C	C23H42N4O5	454.6	32	0	0.83	15	6	4	128.05	141.83	3.2	-1	1.12	0.58	2.13	1.21	-1.04	41.6	0.0915	Very soluble	-1.49	14.6	0.0322	Very soluble	-2.76	0.792	0.00174	Soluble	High	No	Yes	No	No	No	No	No	-9.78	0	1	2	1	0	0.55	0	0	2	4.44
87	LLLP	CC(C[C@@H](C(=O)N[C@H](C(=O)N1CCC[C@H]1C(=O)O)CC(C)C)NC(=O)[C@H](	C23H	454.	32	0	0.83	15	6	4	128.	141.	2.26	-1.1	1.12	0.58	2.13	0.98	-0.9	54	0.119	Very	-1.3	22.5	0.0495	Very so	-2.76	0.792	0.00174	Solubl	High	No	Yes	No	No	No	No	No	-9.9	0	1	2	1	0	0.55	0	0	2	4.45

		CC(C)C)N)C	42N4O5	6							05	83		8					3			soluble	1			luble				e									1										
88	LFSL	OC[C@@H](C(=O)N[C@H](C(=O)O)CC(C)C)NC(=O)[C@@H](NC(=O)[C@H](CC(C)C)N)Cc1ccccc1	C24H38N4O6	478.58	34	6	0.58	17	7	6	127.77	170.85	1.84	-2.13	0.18	0.17	1.94	0.4	-0.47	161	0.336	Very soluble	-0.93	56.4	0.118	Very soluble	-4.14	0.0344	0.000719	Moderately soluble	Low	No	Yes	No	No	No	No	No	-10.73	1	1	2	1	4	0.55	0	0	2	4.36
89	VAWF	O=C([C@H](Cc1c[nH]c2c1cccc2)NC(=O)[C@@H](NC(=O)[C@H](C(C)C)N)C)N[C@H](C(=O)O)Cc1ccccc1	C28H35N5O5	521.61	38	15	0.36	15	6	6	143.73	166.41	2.24	-0.29	1.5	0.48	3.15	1.42	-2.19	3.34	0.00641	Soluble	-2.74	0.939	0.0018	Soluble	-6.83	0.000777	0.0000149	Poorly soluble	Low	No	Yes	No	No	No	No	No	-9.69	2	3	2	1	2	0.17	0	0	2	4.55
90	WVL	CC(C[C@@H](C(=O)O)NC(=O)[C@H](C(C)C)NC(=O)[C@H](Cc1c[nH]c2c1cccc2)N)C	C22H32N4O4	416.51	30	9	0.5	12	5	5	116.24	137.31	2.96	-0.84	1.79	0.68	2.8	1.48	-1.32	19.8	0.0475	Very soluble	-1.56	11.4	0.0274	Very soluble	-4.81	0.0648	0.0000156	Moderately soluble	High	No	Yes	No	No	No	No	No	-9.44	0	0	1	1	0	0.55	0	0	2	3.91
91	LVW	CC(C[C@@H](C(=O)N[C@H](C(=O)N[C@H](C(=O)O)Cc1c[nH]c2c1cccc2)C(C)C)N)C	C22H32N	416.51	30	9	0.5	12	5	5	116.24	137.31	2.29	-0.84	1.79	0.68	2.8	1.35	-1.32	19.8	0.0475	Very sol	-1.56	11.4	0.0274	Very soluble	-4.81	0.0648	0.0000156	Moderately	High	No	Yes	No	No	No	No	No	-9.44	0	0	1	1	0	0.55	0	0	2	3.94

			4O4																			uble								soluble																			
92	LWV	CC(C[C@@H](C(=O)N[C@H](C(=O)N[C@H](C(=O)O)C(C)C)Cc1c[nH]c2c1cccc2)N)C	C22H32N4O4	416.51	30	9	0.5	12	5	5	116.24	137.31	1.95	-0.84	1.79	0.68	2.8	1.28	-1.32	19.8	0.0475	Very soluble	-1.56	11.4	0.0274	Very soluble	-4.81	0.00648	0.0000156	Moderately soluble	High	No	Yes	No	No	No	No	No	-9.44	0	0	1	1	0	0.55	0	0	2	3.95
93	WLM	CSCC[C@@H](C(=O)O)NC(=O)[C@@H](NC(=O)[C@H](Cc1c[nH]c2c1cccc2)N)CC(C)C	C22H32N4O4S	448.58	31	9	0.5	14	5	5	123.83	162.61	1.96	-0.34	1.89	0.68	3.09	1.46	-1.7	9	0.0201	Very soluble	-2.61	1.09	0.00244	Soluble	-5.26	0.00246	0.00000548	Moderately soluble	Low	No	Yes	No	No	No	No	No	-9.28	0	0	2	1	1	0.55	0	0	2	4.19
94	WYL	CC(C[C@@H](C(=O)O)NC(=O)[C@@H](NC(=O)[C@H](Cc1c[nH]c2c1cccc2)N)Cc1ccc(cc1)O)C	C26H32N4O5	480.56	35	15	0.35	13	6	6	133.13	157.54	2.17	-0.06	2.09	0.79	3.23	1.64	-2.24	2.76	0.00574	Soluble	-2.8	0.766	0.00159	Soluble	-6.27	0.00026	0.000000541	Poorly soluble	Low	No	Yes	No	No	No	No	No	-9.27	1	2	2	1	2	0.55	0	0	2	4.13
95	LAWL	CC(C[C@@H](C(=O)N[C@H](C(=O)N[C@H](C(=O)N[C@H](C(=O)O)CC(C)C)Cc1c[nH]c2c1cccc2)C)N)C	C26H39N5O5	501.62	36	9	0.54	16	6	6	138.47	166.41	2.13	-0.73	1.69	0.3	3.12	1.3	-1.62	12.1	0.024	Very solubl	-2.29	2.58	0.00515	Soluble	-5.56	0.00138	0.00000275	Moderately solubl	Low	No	Yes	No	No	No	No	No	-9.88	2	3	2	1	3	0.17	0	0	2	4.61

																						e								e																			
96	FWV	N[C@H](C(=O)N[C@H](C(=O)N[C@H](C(=O)O)C(C)C)Cc1c[nH]c2c1cccc2)Cc1ccccc1	C25H30N4O4	450.53	33	15	0.32	12	5	5	126.3	137.31	1.82	-0.04	1.99	1.09	3.27	1.63	-2.15	3.17	0.00704	Soluble	-2.39	1.82	0.00404	Soluble	-6.47	0.000153	0.0000034	Poorly soluble	High	No	Yes	No	No	No	Yes	No	-9.08	0	0	1	1	0	0.55	0	0	2	4
97	WLTL	CC(C[C@@H](C(=O)N[C@H](C(=O)N[C@H](C(=O)O)CC(C)C)[C@H](O)C)NC(=O)[C@H](Cc1c[nH]c2c1cccc2)N)C	C27H41N5O6	531.64	38	9	0.56	17	7	7	144.44	186.64	3.26	-1.35	1.05	-0.26	2.79	1.1	-1.34	24.4	0.0458	Very soluble	-2.07	4.53	0.00852	Soluble	-5	0.000532	0.000001	Moderately soluble	Low	No	Yes	No	No	No	No	No	-10.5	3	3	2	1	3	0.17	0	0	2	4.88
98	LLW	CC(C[C@@H](C(=O)N[C@H](C(=O)O)Cc1c[nH]c2c1cccc2)NC(=O)[C@H](CC(C)C)N)C	C23H34N4O4	430.54	31	9	0.52	13	5	5	121.05	137.31	2.35	0.18	2.18	0.9	3.21	1.77	-1.98	4.51	0.0105	Very soluble	-2.62	1.03	0.00239	Soluble	-5.2	0.00272	0.0000632	Moderately soluble	High	No	Yes	No	No	No	No	No	-8.8	0	0	1	1	0	0.55	0	0	2	4.06
99	LFF	CC(C[C@@H](C(=O)N[C@H](C(=O)N[C@H](C(=O)O)Cc1ccccc1)Cc1ccccc1)N)C	C24H31N3O4	425.52	31	12	0.38	13	5	4	119.25	121.52	2.61	-0.29	1.9	1.95	3.11	1.85	-1.72	8.03	0.0189	Very soluble	-1.8	6.71	0.0158	Very soluble	-6.01	0.000414	0.0000973	Poorly soluble	High	No	Yes	No	No	No	Yes	Yes	-9.1	0	0	1	0	0	0.55	0	0	2	3.74
10	FL	CC(C[C@@H](C(=O)N[C@H](C(=O)O)Cc	C2	42	31	12	0.	13	5	4	11	12	2.	-0	1.	1.	3.1	1.6	-1	13.9	0.03	Ve	-1	16.6	0.03	Ver	-6.	0.000	0.000	Po	Hi	No	Ye	No	No	No	Yes	Yes	-9	0	0	1	0	0	0.55	0	0	2	3.74

0	F	1ccccc1)NC(=O)[C@H](Cc1ccccc1)N)C	4H31N3O4	5.52			38				9.25	1.52	04	.67	9	95	1	6	.48		28	rysoluble	.41		91	ysoluble	01	414	000973	orlysoluble	gh		s						.37										
101	LFVL	CC(C[C@@H](C(=O)N[C@H](C(=O)N[C@H](C(=O)N[C@H](C(=O)O)CC(C)C)C(C)C)Cc1ccccc1)N)C	C26H42N4O5	490.64	35	6	0.62	17	6	5	136.23	150.62	2.06	-0.25	1.84	1.35	3.2	1.64	-1.73	9.15	0.0187	Very soluble	-2.45	1.72	0.00351	Soluble	-5.12	0.0037	0.0000753	Moderately soluble	Low	No	Yes	No	No	No	No	No	-9.47	0	3	2	1	2	0.55	0	0	2	4.48
102	LLLL	CC(C[C@@H](C(=O)N[C@H](C(=O)N[C@H](C(=O)O)CC(C)C)CC(C)C)NC(=O)[C@H](CC(C)C)N)C	C24H46N4O5	470.65	33	0	0.83	18	6	5	130.97	150.62	3.39	0.13	2.04	1.19	3.13	1.98	-1.65	10.5	0.0223	Very soluble	-2.85	0.666	0.00142	Soluble	-3.86	0.0656	0.000139	Soluble	Low	No	Yes	No	No	No	No	No	-9.08	0	2	2	1	2	0.55	0	0	2	4.6
103	WLVL	CC(C[C@@H](C(=O)N[C@H](C(=O)N[C@H](C(=O)O)CC(C)C)C(C)C)NC(=O)[C@H](Cc1c[nH]c2c1cccc2)N)C	C28H43N5O5	529.67	38	9	0.57	17	6	6	148.08	166.41	3.02	0.23	2.32	0.7	3.8	2.02	-2.32	2.52	0.00476	Soluble	-3.28	0.275	0.000519	Soluble	-5.96	0.000575	0.00000109	Moderately soluble	Low	No	Yes	No	No	No	No	No	-9.37	2	3	2	1	3	0.17	0	0	2	4.81
104	LWF	CC(C[C@@H](C(=O)N[C@H](C(=O)N[C@H](C(=O)O)Cc1ccccc1)Cc1c[nH]c2c1ccc	C26H	464.	34	15	0.35	13	5	5	131.	137.	2.37	0.28	2.38	1.3	3.69	2	-2.3	2	0.00431	Solu	-2.7	0.875	0.00188	Solubl	-6.86	0.000643	0.000001	Poorly	High	No	Yes	No	No	No	Yes	No	-8.9	0	1	1	1	0	0.55	0	0	2	4.12

		c2)N)C	32N4O4	56							11	31							7			ble	3			e			38	soluble									3										
105	LFW	CC(C[C@@H](C(=O)N[C@H](C(=O)N[C@H](C(=O)O)Cc1c[nH]c2c1cccc2)Cc1ccccc1)N)C	C26H32N4O4	464.56	34	15	0.35	13	5	5	131.11	137.31	2.16	0.28	2.38	1.3	3.69	1.96	-2.37	2	0.00431	Soluble	-2.73	0.875	0.00188	Soluble	-6.86	0.0000643	0.000000138	Poorly soluble	High	No	Yes	No	No	No	Yes	No	-8.93	0	1	1	1	0	0.55	0	0	2	4.11
106	IPI	CC[C@@H]([C@@H](C(=O)O)NC(=O)[C@@H]1CCCN1C(=O)[C@H]([C@H](CC)C)N)C	C17H31N3O4	341.45	24	0	0.82	10	5	3	96.21	112.73	2.13	-1.04	0.59	0.48	1.11	0.65	-0.64	77.9	0.228	Very soluble	-0.84	49.4	0.145	Very soluble	-1.58	9.02	0.0264	Soluble	High	No	Yes	No	No	No	No	No	-9.12	0	0	0	0	0	0.55	0	0	1	3.81
